# Comprehensive analysis and exploratory design of graphene-based subharmonic mixers operating at the gigahertz band

**DOI:** 10.1186/s11671-025-04221-x

**Published:** 2025-03-14

**Authors:** M. C. Pardo, F. Pasadas, A. Medina-Rull, M. G. Palomo, S. Ortiz-Ruiz, E. G. Marin, A. Godoy, F. G. Ruiz

**Affiliations:** Pervasive Electronics Advanced Research Laboratory (PEARL), Departamento de Electrónica y Tecnología de Computadores, Universidad de Granada, 18071 Granada, Spain

**Keywords:** Graphene field-effect transistor, Ambipolar electronics, Subharmonic mixing, Radio-frequency, Large-signal S-parameters, Impedance matching

## Abstract

Ambipolar conductance in graphene field-effect transistors (GFETs), and in particular their quasi-quadratic I–V transfer characteristic, makes these devices excellent candidates for exploiting subharmonic mixing at high frequencies. Several realizations have already demonstrated the ability of GFETs to compete with, or even improve, state-of-the-art mixers based on traditional technologies. Nonetheless, a systematic analysis of the influence on performance of both circuit design and technological aspects has not been conducted yet. In this work, we present a comprehensive assessment of the conversion losses by means of applying radio-frequency circuit design techniques in terms of filtering and matching, along with the impact stemming from physical and geometric variations of a fabricated graphene technology.

## Introduction

Research on 2D materials has garnered significant interest over the past two decades, with graphene emerging as the most promising candidate for high-frequency applications [1], primarily due to its exceptional carrier mobility [2, 3]. Some examples of the main advancements can be found among radio-frequency (RF) power detection applications [4, 5], antenna arrays [6], phase shifters [7], frequency multipliers [8–10], low noise amplifiers [11, 12], modulators [13] and even THz absorbers [14, 15]. Furthermore, the ambipolar electrical response of GFETs, i.e. their symmetric V-shaped transfer characteristics (${I}_{\text{DS}}$ versus ${V}_{\text{GS}}$) around the point of minimum conductivity, namely, the Dirac voltage (${V}_{\text{Dirac}}$), enables the exploration of new functionalities as well as the redesign and simplification of conventional RF circuits [16, 17].

**Fig. 1:**
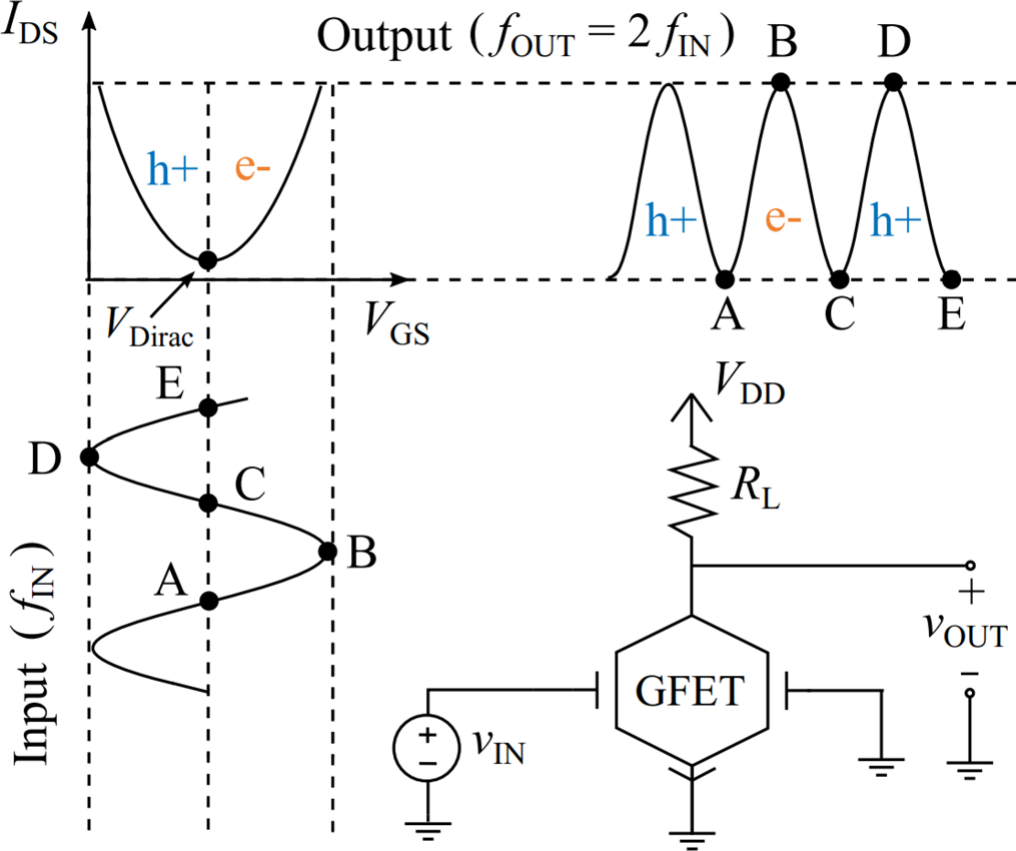
Working principle of a GFET-based frequency doubler, relying on the intrinsic graphene ambipolar conduction. The same concept is leveraged in subharmonic mixers to generate a higher-frequency component at twice the LO frequency when gate biasing is centered at ${V}_{\text{Dirac}}$

In the particular realm of mixers, the low transconductance of GFETs still hinders the deployment of active architectures with comparable performance to conventional technologies [1, 16]. The scenario is more favorable with subharmonic resistive mixers (taking advantage from the frequency doubling phenomenon enabled by graphene quadratic ${I}_{\text{DS}}$—${V}_{\text{GS}}$ response, see Fig. 1), which have demonstrated to be on par with state-of-the-art mixing performance [18–21]. Subharmonic mixers are especially convenient at high frequencies since they relax the frequency requirement of the high-power local oscillator (LO) source [22]. In addition, the larger frequency gap between the RF and the LO signals that generate a given intermediate frequency (IF) signal also eases the filter design for preventing the LO leakage through the gate-drain capacitance to the drain terminal, a concern that becomes more pronounced as the frequency is raised. Furthermore, the utilization of the GFET as the main building block of subharmonic mixers considerably reduces the complexity of the system as it avoids the need of i) two out-of-phase LO signals through a hybrid coupler that doubles the number of transistors [23, 24], or ii) a pair of anti-parallel diodes [25]; simplifying the fabrication process in an eventual integrated circuit (IC) implementation. Not only that, but this GFET mixer topology also features zero DC power consumption as the drain is left unbiased in the resistive mixer configuration. This characteristic paves the way for the ulterior development of ultra-low-power transceivers targeting the deployment of ubiquitous wireless communication systems in the IoT scene.

Motivated by these auspicious features, this work focuses on two well-defined objectives: i) To realize an optimized design of a GFET-based subharmonic resistive mixer, particularly concerning the power ratio of the output IF signal to the input RF signal, namely, conversion losses (CL). With this, our aim is to contribute with a robust and systematic methodology for designing single-GFET mixers that also applies to other mixer architectures, incorporating innovative circuit-level techniques, related to the selective filtering of undesired frequency components and the matching between ports. ii) To analyze the impact of the GFET geometrical and technological parameters on the mixer performance. This analysis is particularly relevant due to the present challenges in achieving uniformity and technological control in GFETs [1], which complicate the development of graphene circuits and consequently impact in the progress of its Technology Readiness Level (TRL).

The study is organized as follows. The computer-aided design (CAD) tool based on fabricated GFET-technology is presented in Sect. 2. In Sect. 3, we outline the mixer design using RF techniques such as selective filtering and an approach for simultaneous conjugate matching the nonlinear circuit operating at different fundamental frequencies. Then, by using the proposed design, in Sect. 4 we undertake different performance predictions spanning critical GFET technological and geometrical parameters to assess its outcome through the analysis of CL. Finally, the main conclusions are drawn in Sect. 5.

## Methods

The experimental results of the top-gated GFET technology reported in [19] are considered here as a reference to benchmark the results achieved by simulation, ensuring that the design process is feasible and could be developed into a manufactured prototype. It consists of an exfoliated graphene flake (1 μm-long × 20 μm-wide) with 25-nm Al_2_O_3_ as gate dielectric.

The main technological and material parameters are collected in Table 1, directly extracted from the fabricated devices reported in [19], where $\mu$ stands for the field-independent effective carrier mobility (considered identical for both electrons and holes); ${V}_{\text{g}0}$ is the gate offset voltage comprising the work-function difference between the gate and the graphene channel as well as the effect of additional fixed charge owing to impurities or doping; ${n}_{0}$ is the residual mobile charge density in the graphene channel associated to electron/hole puddles; ${\rho }_{\text{c}}$ is the metal-graphene contact resistivity (considered the same at the source and drain channel edges); ${\rho }_{\text{g}}$ is the parasitic gate pad resistivity; ${L}_{\text{g}}$ and ${W}_{\text{g}}$ are the gate length and width, respectively; and ${t}_{\text{ox}}$ and ${\epsilon }_{\text{ox}}$ are the gate oxide thickness and relative permittivity, respectively. Further information about the meaning of the CAD tool parameters can be found elsewhere [17, 26].

**Table 1:** Input parameters for the CAD tool used to reproduce the measured data of the GFET reported in [19]

Parameter	Value	Parameter	Value
$\mu$	2100 cm^2^/Vs	${L}_{\text{g}}$	1 μm
${V}_{\text{g}0}$	1 V	${W}_{\text{g}}$	20 μm
${n}_{0}$	9.9 × 10^11^ cm^−2^	${t}_{\text{ox}}$	25 nm
${\rho }_{\text{c}}$	560 Ω × μm	${\epsilon }_{\text{ox}}$	8.4
${\rho }_{\text{g}}$	21 Ω × μm		

$\mu$ is the effective carrier mobility; ${V}_{\text{g}0}$ is the gate offset voltage; ${n}_{0}$ is the residual charge density associated to electron/hole puddles; ${\rho }_{\text{c}}$ is the metal-graphene contact resistivity; ${\rho }_{\text{g}}$ is the parasitic gate pad resistivity; ${L}_{\text{g}}$ and ${W}_{\text{g}}$ are the gate length and width, respectively; and ${t}_{\text{ox}}$ and ${\epsilon }_{\text{ox}}$ are the gate oxide thickness and relative permittivity, respectively

**Fig. 2:**
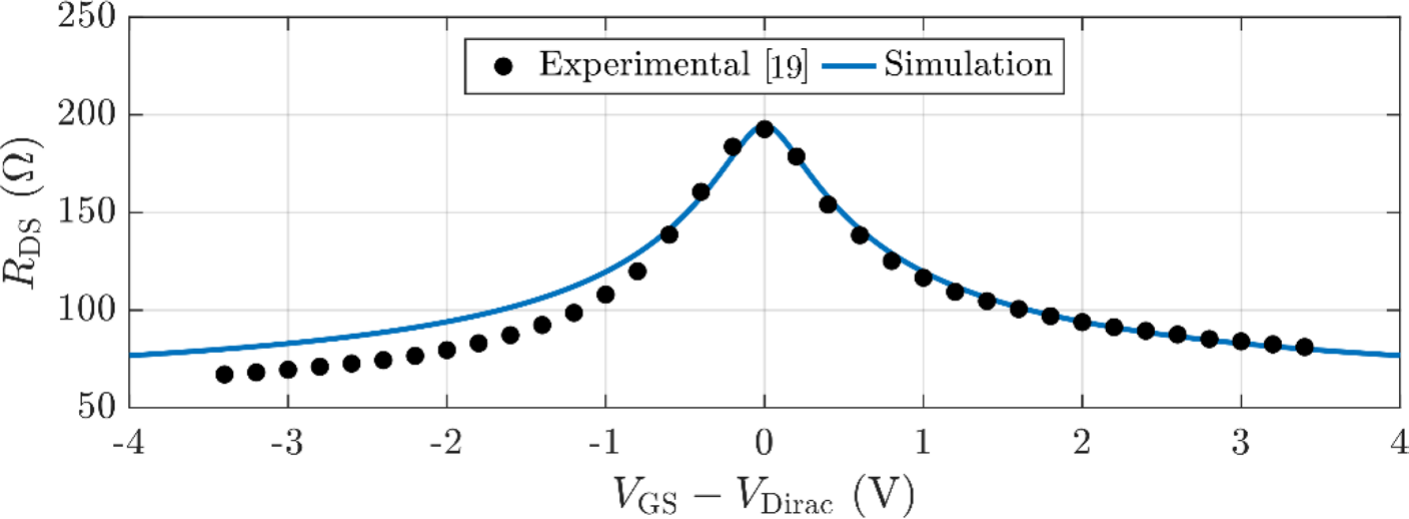
Drain to source resistance, ${R}_{\text{DS}}={V}_{\text{DS}}/{I}_{\text{DS}}$, versus overdrive gate voltage ${V}_{\text{GS}}-{V}_{\text{Dirac}}$

Note that these parameters will be used by default for the performance prediction throughout this work, except otherwise stated. To proceed with the predictive study of GFET subharmonic mixers performance, we consider the large-signal compact model developed by some of the authors in [26] and [27], and embedded into Keysight Advanced Design Systems (ADS©). Firstly, the model is validated at the device and circuit levels, combining experimental data and simulation. In particular, the DC characteristic ${R}_{\text{DS}}-{V}_{\text{GS}}$ of the considered GFET is compared against experimental measurements in Fig. 2, demonstrating excellent agreement in a wide range of bias around the Dirac point. In the following, we present and validate the GFET-based subharmonic mixer circuit.

## Theory and design of GFET-based subharmonic mixers

**Fig. 3:**
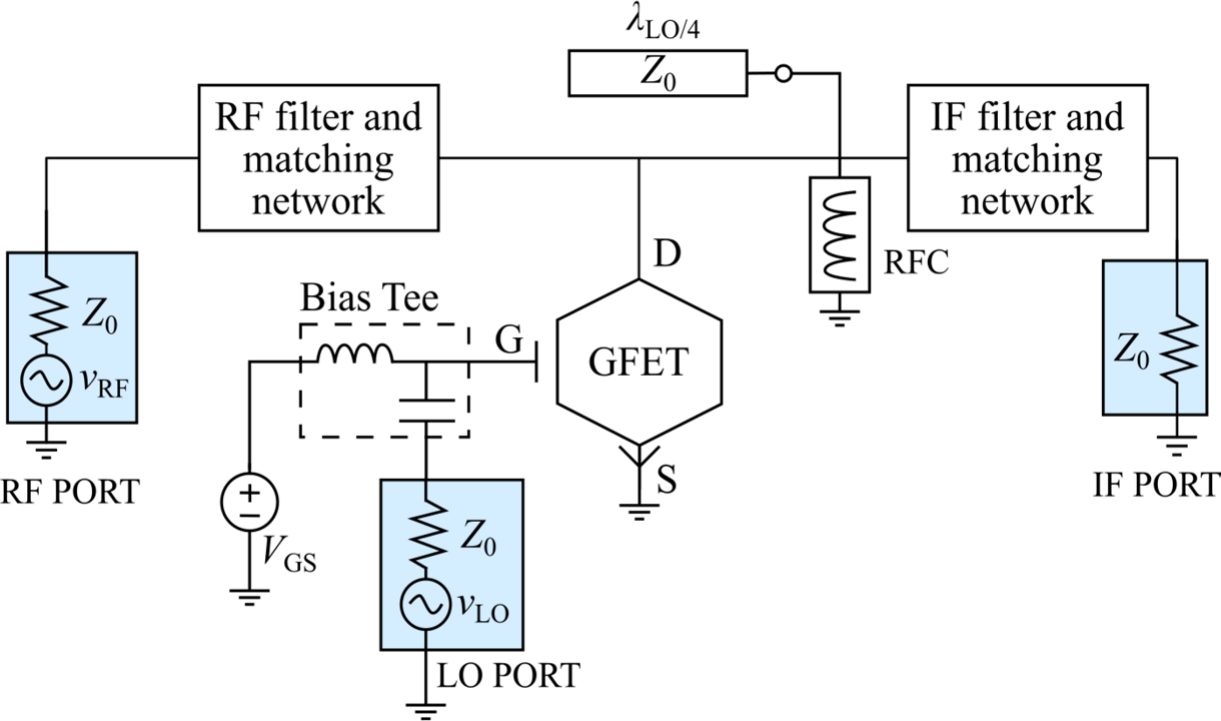
Schematic showing the designed single-GFET subharmonic mixer, including a LO rejection filter. The RFC ensures zero drain voltage, while ${V}_{\text{GS}}={V}_{\text{Dirac}}$. A bias tee is added to insert the LO signal, as well as low and high pass filters to isolate IF and RF components at the drain of the GFET

The schematic of the single-GFET mixer design proposed and studied in this work is depicted in Fig. 3. The resistive configuration implies that the LO signal is connected to the gate terminal of the GFET, whereas the RF and the IF signals are inserted and extracted, respectively, through the drain terminal. The input ports have associated frequencies ${f}_{\text{LO}}$ and ${f}_{\text{RF}}$, so that the expected down-converted signal is ${f}_{\text{IF}}={f}_{\text{RF}}-2{f}_{\text{LO}}$, due to the frequency doubling achieved when ${V}_{\text{GS}}={V}_{\text{Dirac}}$ (cf. working principle in Fig. 1). As displayed in the schematic, a DC voltage source at the drain is not required, and an RF choke (RFC) guarantees ${V}_{\text{DS}}=0$ V, preventing DC power consumption. It is worth to note that a DC voltage source at the gate would neither be necessary if ${V}_{\text{Dirac}}=0$ V. In a three-terminal GFET, for ${V}_{\text{DS}}=0$ V, the Dirac voltage lies at ${V}_{\text{Dirac}}={V}_{\text{g}0}$, which depends on the work-function difference between the gate metal and the graphene channel, as well as the possible presence of additional charges due to impurities or uncontrolled doping [28]. Indeed, intentional doping could be harnessed for GFET-based subharmonic mixing with no DC sources by properly tuning ${V}_{\text{Dirac}}=0$ V. Naturally, this simple mixer topology requires proper filtering and impedance matching to mitigate port-to-port coupling, primarily caused by the LO large-signal, and to achieve the cleanest possible IF signal at its output port. These functions are satisfied by the blocks shown in the schematic, that will be addressed and detailed in the following subsections.

### Selective rejection of spurious responses

A common rule in the design of single-FET resistive mixers (where the isolation between ports is weaker than that of the balanced structures) is to short-circuit the LO signal at the drain terminal [12, 17]. This feature becomes essential when the considered frequency range is high enough so that significant gate-to-drain coupling can be expected through the FET intrinsic gate-to-drain capacitance. A noteworthy feature in the design presented in Fig. 3 is the inclusion of an LO rejection filter, consisting in an open-loaded ${\lambda }_{\text{LO}}/4$ stub, to reduce the distortion of the output mixed signal without practically affecting neither the RF component nor the desired IF.

**Fig. 4:**
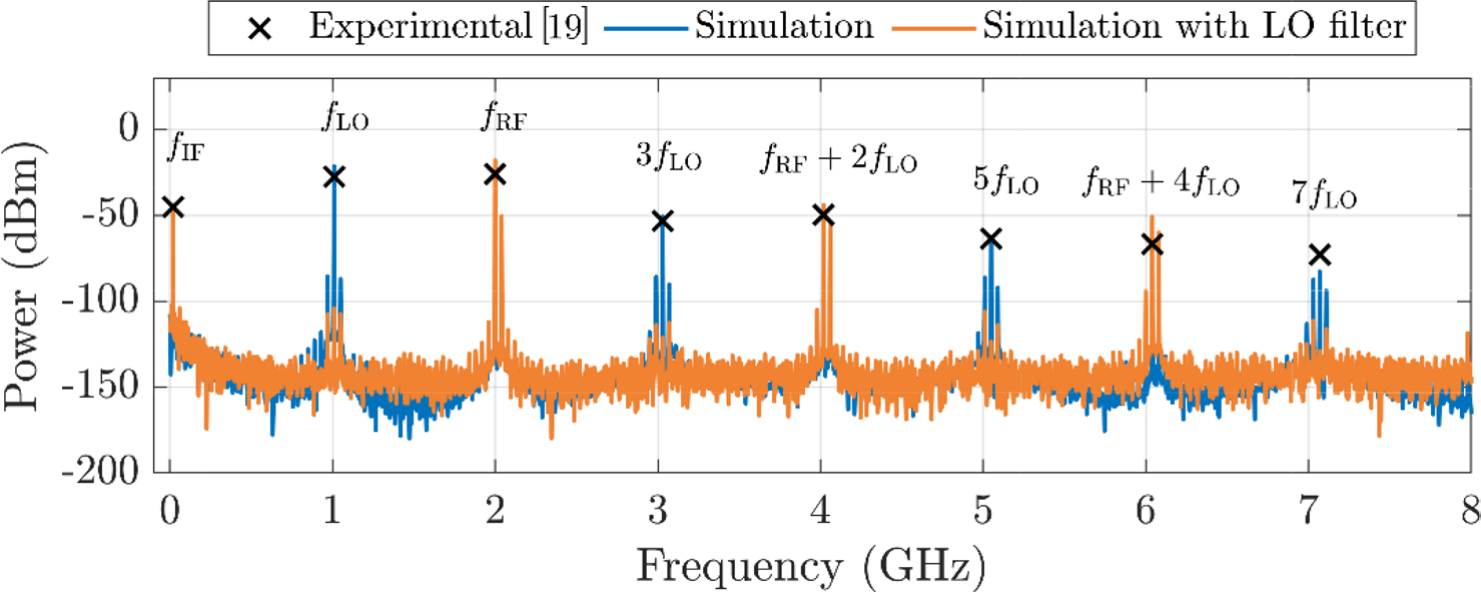
Power spectral distribution obtained at the drain of the GFET, with ${P}_{\text{LO}}=15$ dBm and ${P}_{\text{RF}}=-20$ dBm. Experimental data from [19] (crosses) and simulation, without (blue line) and with (orange line) the LO rejection filter, are compared. The simulation of the original topology properly fits the experimental data, while the modified circuit shows rejection of components involving odd LO harmonics, resulting in a less distorted mixed signal

Figure 4 illustrates the resulting mixer output spectra for ${f}_{\text{LO}}=1.01$ GHz and ${f}_{\text{RF}}=2$ GHz (so that ${f}_{\text{IF}}=20$ MHz), as derived from time-domain simulations of the topology shown in Fig. 3. Experimental data from [19] and the simulated result prior to the addition of the LO rejection filter reveal very similar power magnitudes, validating the model to an excellent agreement also at the RF regime. Furthermore, when considering the LO rejection filter, not only is the short-circuit of the fundamental LO frequency achieved, but, because of the periodic behavior of the distributed element, this short occurs at every odd multiple (${f}_{\text{LO}}$, $3{f}_{\text{LO}}$, …). Conversely, even-order components ($2{f}_{\text{LO}}$, $4{f}_{\text{LO}}$, …) face an open-circuit impedance, so they are not affected by the filter. Due to the frequency proximity of ${f}_{\text{RF}}$ and $2{f}_{\text{LO}}$, RF harmonics also remain unaltered. This aspect is critical to allow maximum RF power feed to the transistor to participate in the mixing process resulting in both unchanged CL and a less distorted mixed signal at the same time.

### Simultaneous conjugate match of RF and IF ports

The inclusion of matching networks in mixers designs is essential in order to reduce power losses, especially in subharmonic topologies, where CL are usually higher than in fundamental architectures [29]. This task entails a challenging endeavor, mainly because of two key factors: 1) the inherent multi-frequency operation of mixers, resulting from the generation of numerous intermodulation frequency components, that precludes the correct loading conditions for all of them; and 2) the large-signal operation, that prevents the characterization in terms of small-signal parameters and therefore the direct application of the design theory associated with them.

To tackle this challenge, designers have sought, on the one hand, to minimize CL by terminating the idler frequencies at the RF and IF ports [22, 30, 31]. However, this approach calls for the introduction of more degrees of freedom into the circuit (to deal with these components impedance terminations) leading to increased complexity. Other authors have relied on the use of load-pull techniques [32, 33], but without offering a theoretical support to determine the optimal impedances. On the other hand, few authors have addressed the appropriate termination of the RF and IF ports at their respective RF and IF frequencies, pursuing conjugate impedance matching [34]. This approach represents the optimal solution when dealing with two-port linear time-invariant (LTI) networks [35, 36] but requires the adoption of some approximations for its application in the nonlinear and multifrequency nature of mixers. Here we will seek to attain a feasible solution to the nonlinear and multifrequency conjugate matching problem, subsequently assessing its validity.

To do so, we first consider only two frequencies in the circuit for the purpose of impedance matching: the RF frequency and the IF frequency. These two signals play a fundamental role in determining the resulting CL of the mixer. Thus, generating optimal loading conditions for them (to improve transmission between their ports and minimize reflections) becomes crucial. The impedance mismatch of the LO port is less relevant [25], as long as we are able to compensate for the LO power waste to achieve optimum CL.

**Fig. 5:**
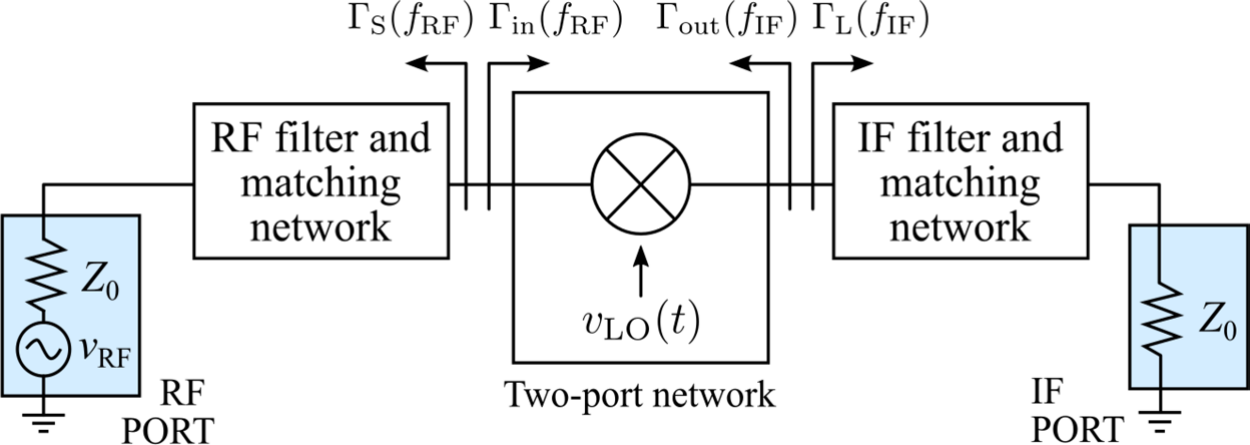
Representation of a mixer as a quasi-linear time-variant two-port network, aiming at the impedance matching realization. The transistor, DC bias and LO source are included in the two-port network

Next, we will treat the circuit as a two-port system, which embeds the local oscillator, as shown in Fig. 5. In this way, the two-port network can be considered quasi-linear, since both ports (RF and IF) are subjected to small-signal excitations. Even so, we will characterize the system using large-signal S-parameters (LSSPs). Unlike small-signal S-parameters, which are derived from the linearization of a circuit under small-signal excitation, LSSPs are obtained through a harmonic balance (HB) simulation of the nonlinear circuit. Since HB is a large-signal simulation technique, LSSPs are power-dependent, making them suitable for evaluating the accuracy of the quasi-linear two-port approximation, particularly as the LO power level increases. Another motivating factor for employing LSSPs is their ability to represent the relationship between incident and reflected power levels at various frequencies, as required in the two-port system under consideration. In the following, S-parameters are defined as follows

1 $${S}_{11}= \frac{{b}_{\text{RF}}}{{a}_{\text{RF}}} =\left|{S}_{11}\right|\angle {\phi }_{b,\text{RF}}-{\phi}_{a,\text{RF}}$$

2 $${S}_{12}= \frac{{b}_{\text{RF}}}{{a}_{\text{IF}}} =\left|{S}_{12}\right|\angle 2\pi \left({f}_{\text{RF}}-{f}_{\text{IF}}\right)t+{\phi }_{\text{b,RF}}-{\phi }_{\text{a,IF}}$$

3 $${S}_{21}= \frac{{b}_{\text{IF}}}{{a}_{\text{RF}}} =\left|{S}_{21}\right|\angle 2\pi \left({f}_{\text{IF}}-{f}_{\text{RF}}\right)t+{\phi }_{\text{b,IF}}-{\phi }_{\text{a,RF}}$$

4 $${S}_{22}= \frac{{b}_{\text{IF}}}{{a}_{\text{IF}}} =\left|{S}_{22}\right|\angle {\phi }_{\text{b,IF}}-{\phi }_{\text{a,IF}},$$

where ${a}_{\text{RF}}$ and ${b}_{\text{RF}}$ are the incident and reflected power waves at the RF port at ${f}_{\text{RF}}$; ${a}_{\text{IF}}$ and ${b}_{\text{IF}}$ are the incident and reflected power waves at the IF port at ${f}_{\text{IF}}$; and ${\phi }_{\text{a},\text{RF}}$, ${\phi }_{\text{b},\text{RF}}$, ${\phi }_{\text{a},\text{IF}}$, and ${\phi }_{\text{b},\text{IF}}$ are the phases associated with these waves.

Despite ${S}_{12}$ and ${S}_{21}$ are time-dependent quantities, it can be mathematically proven that the optimal reflection coefficients towards the generator and the load, ${\Gamma }_{\text{MS}}$ and ${\Gamma }_{\text{ML}}$, respectively, are time-independent [34]. This is due to the fact that ${S}_{12}$ and ${S}_{21}$ appear in the ${\Gamma }_{\text{MS}}$ and ${\Gamma }_{\text{ML}}$ equations (see Appendix A) solely in the calculation of $\Delta ={S}_{11}{S}_{22}-{S}_{21}{S}_{12}$, i.e., as a product between them, where the time-dependence is cancelled: $S_{12} S_{21} = \left| {S_{12} } \right|\left| {S_{21} } \right|\angle \Delta \phi_{{{\text{RF}}}} - \Delta \phi_{{{\text{IF}}}}$ and thus $\Delta$ is a time-invariant quantity. Therefore, for a specific LO power level, it is possible to implement the matching networks that satisfy (cf. Fig. 5)

5 $${\Gamma }_{\text{MS}}={\Gamma }_{\text{S}}\left({f}_{\text{RF}}\right)={\Gamma }_{\text{in}}^{*}\left({f}_{\text{RF}}\right)$$

6 $${\Gamma }_{\text{ML}}={\Gamma }_{\text{L}}\left({f}_{\text{IF}}\right)={\Gamma }_{\text{out}}^{*}\left({f}_{\text{IF}}\right)$$

**Fig. 6:**
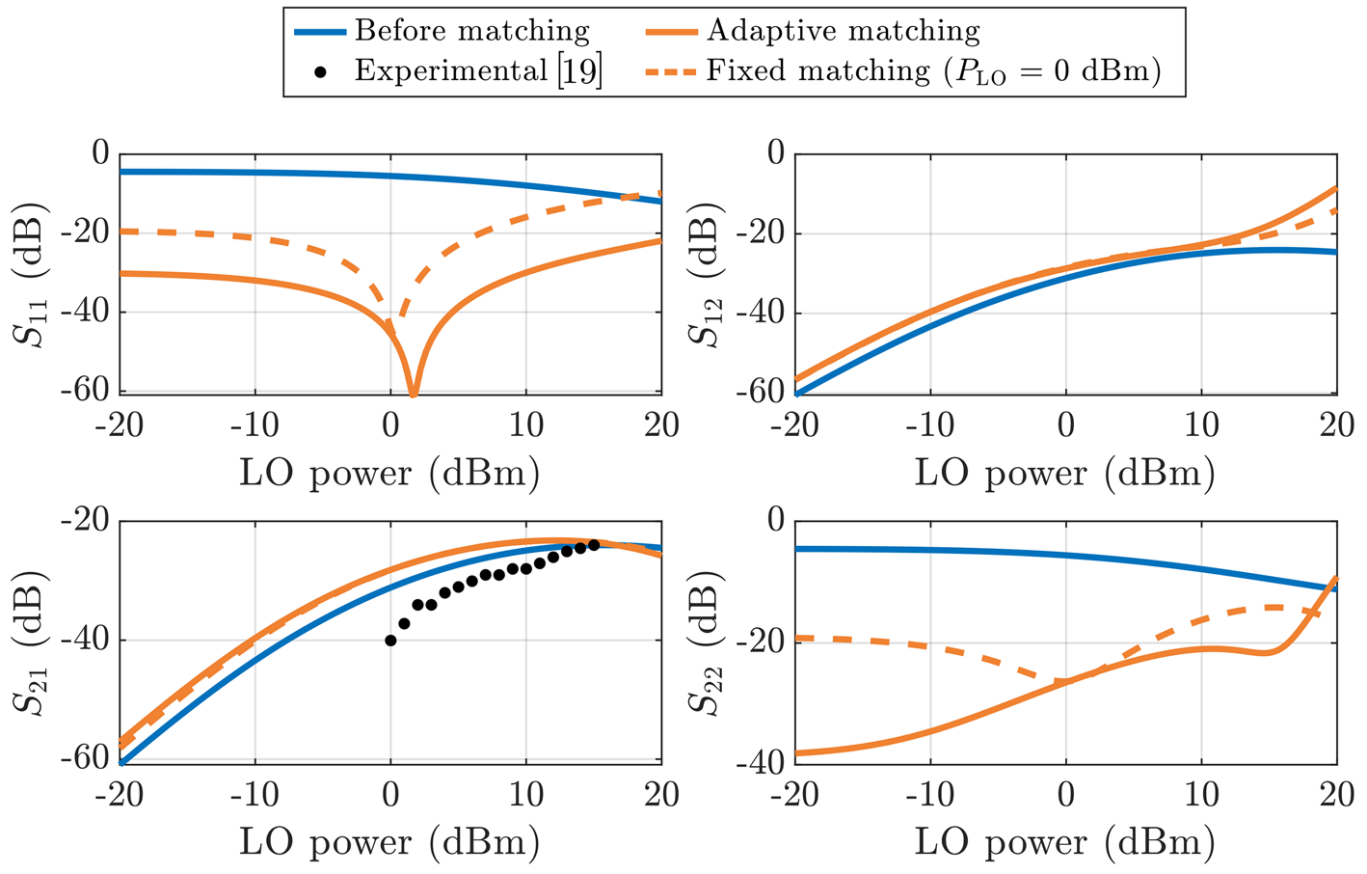
Magnitude of the LSSP before (blue line) and after matching for a fixed input ${P}_{\text{LO}}=0$ dBm (orange dashed line) and when adapting the matching networks for each ${P}_{\text{LO}}$ (orange solid line) following the proposed implementation based on the two-port quasi-linear approximation. Black dots represent the measurement on the fabricated graphene-based subharmonic mixer [19]

Before the implementation of any impedance matching, the LSSP, depicted in blue solid lines in Fig. 6, are utilized to evaluate ${\Gamma }_{\text{MS}}$ and ${\Gamma }_{\text{ML}}$, evidencing large mismatch reflections. Then, L-networks, simultaneously working as high and low pass filters for RF and IF, respectively, are employed to tune them. In a first step, the matching networks are designed for each ${P}_{\text{LO}}$ value (i.e., adaptive matching, orange solid lines in Fig. 6). Based on the results, the reflection parameters (${S}_{11}$ and ${S}_{22}$) exhibit a significant improvement compared to the mismatched case, especially at low power levels. However, despite the matching networks being recalculated for each LO power, at high power levels ${S}_{11}$ and ${S}_{22}$ increase up to the point of reaching magnitudes similar to those without impedance matching (i.e., blue solid lines in Fig. 6). This is due to the fact that the implemented matching networks fail to achieve the expected impedance, as the small-signal approximation no longer holds.

**Fig. 7:**
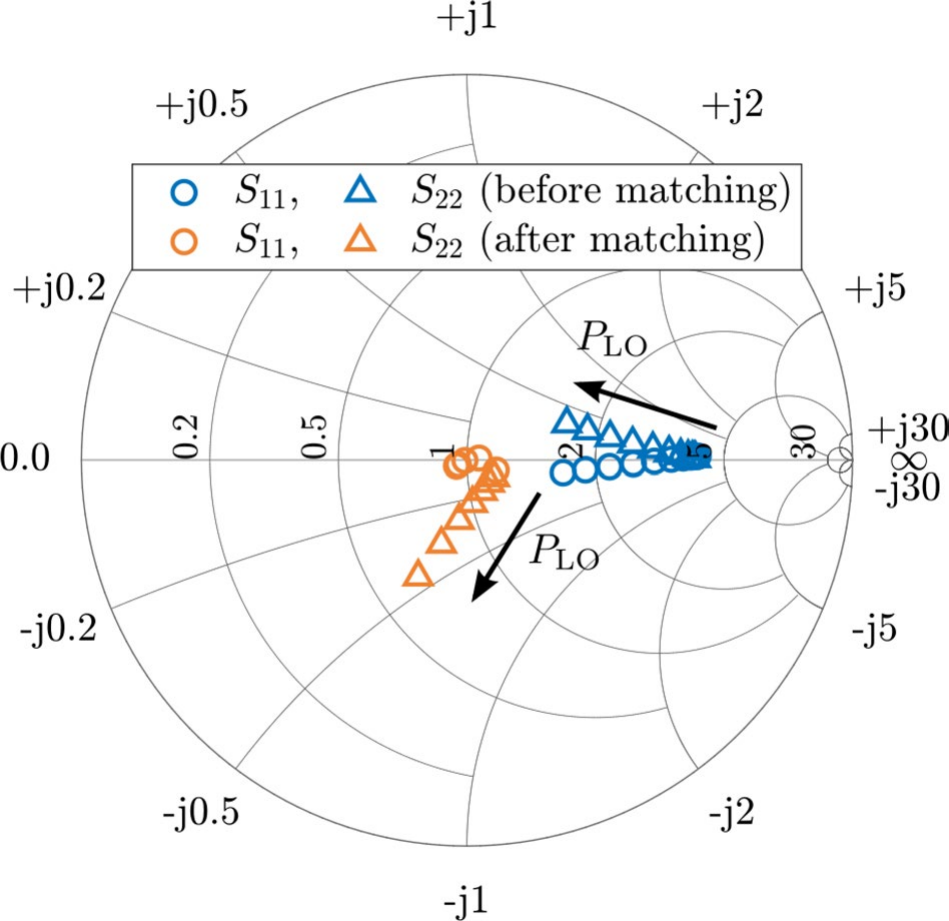
Reflection LSSP before (blue) and after (orange) the introduction of adaptive impedance matching

To gain a deeper understanding of the underlying reason for the operational limitations of this matching approach, Fig. 7 depicts the Smith chart LSSP (${S}_{11}$ and ${S}_{22}$) of the designed mixer before and after the adaptive matching. Both parameters are, after the matching, shifted closer to the center of the chart for low ${P}_{\text{LO}}$, demonstrating the effectiveness of the matching networks. By contrast, as ${P}_{\text{LO}}$ increases, a considerable deviation from the chart center occurs, which is much more noticeable for the reflection associated with the IF port (${S}_{22}$).

Finally, as for a practical implementation, the impact on the LSSP when considering the fixed matching networks designed for ${P}_{\text{LO}}=0\hspace{0.25em}\text{dBm}$ is also analyzed. In this case, ${S}_{11}$ (dashed orange lines in Fig. 6) shows some significant variation for ${P}_{\text{LO}}\ne 0$ dBm, increasing roughly by 20 dB. ${S}_{22}$ remains considerably stable across a wide range of power levels. Remarkably, both magnitudes are kept below − 10 dB throughout the LO power window under test, reaching an acceptable matching for mixing operation. Concerning the transmission parameters (${S}_{12}$ and ${S}_{21}$), they both experience an enhancement (although of much lower magnitude) with matching, as depicted in Fig. 6. In particular,  $\sim$3 dB of improvement is achieved across a fairly wide range of power levels. An interesting observation is that the fixed matching design at ${P}_{\text{LO}}=0\hspace{0.25em}\text{dBm}$ yields essentially the same results as the adaptive case, leading to the conclusion that ${S}_{12}$ and ${S}_{21}$ are mostly insensitive to the variation of ${P}_{\text{LO}}$ at low power levels.

The behavior of losses can be analyzed looking at Fig. 6c, given that they are equivalent to the LSSP $-\left|{S}_{21}\right|$. As extracted from it, the CL can be improved by pumping the gate with higher LO level. However, its power cannot be increased indefinitely, since, at some point, the rectified voltage at the drain of the intrinsic device is enough to generate a significant shift of the transfer characteristic given by ${V}_{\text{Dirac}}={V}_{\text{g}0}+{V}_{\text{DS}}/2$ [28], so that the second-order harmonic of LO is degraded and CL increases. The experimental data from Fig. 6c did not display this regime under the utilized LO power levels, but the simulation demonstrates the deterioration of CL as ${P}_{\text{LO}}$ is higher than 15 dBm.

## Results and discussion

We will explore now the influence of the GFET technological and geometrical variables on the mixer performance, paying particular attention to the CL and the on–off resistance ratio as main figures of merit. Unlike transconductance mixers, the metric that needs to be optimized in a resistive mixer is not the time-dependent transconductance ${g}_{\text{m}}\left(t\right)$, but the time-dependent channel conductance ${G}_{\text{DS}}\left(t\right)$ [25]. This variable is related to the DC ${I}_{\text{DS}}-{V}_{\text{GS}}$ response, as long as the LO signal is able to bias the GFET around ${V}_{\text{GS}}={V}_{\text{Dirac}}$, and thus generate a time-varying resistance ${R}_{\text{DS}}\left(t\right)=1/{G}_{\text{DS}}\left(t\right)$. We will analyze the role of the main technological (i.e. gate offset voltage, effective carrier mobility, contact resistivity, and residual carrier density) and geometrical (gate length and oxide thickness) parameters on the device DC operation by inspecting the ${I}_{\text{DS}}-{V}_{\text{GS}}$ characteristics, and more importantly their eventual impact on the on–off resistance ratio and on the CL.

**Fig. 8:**
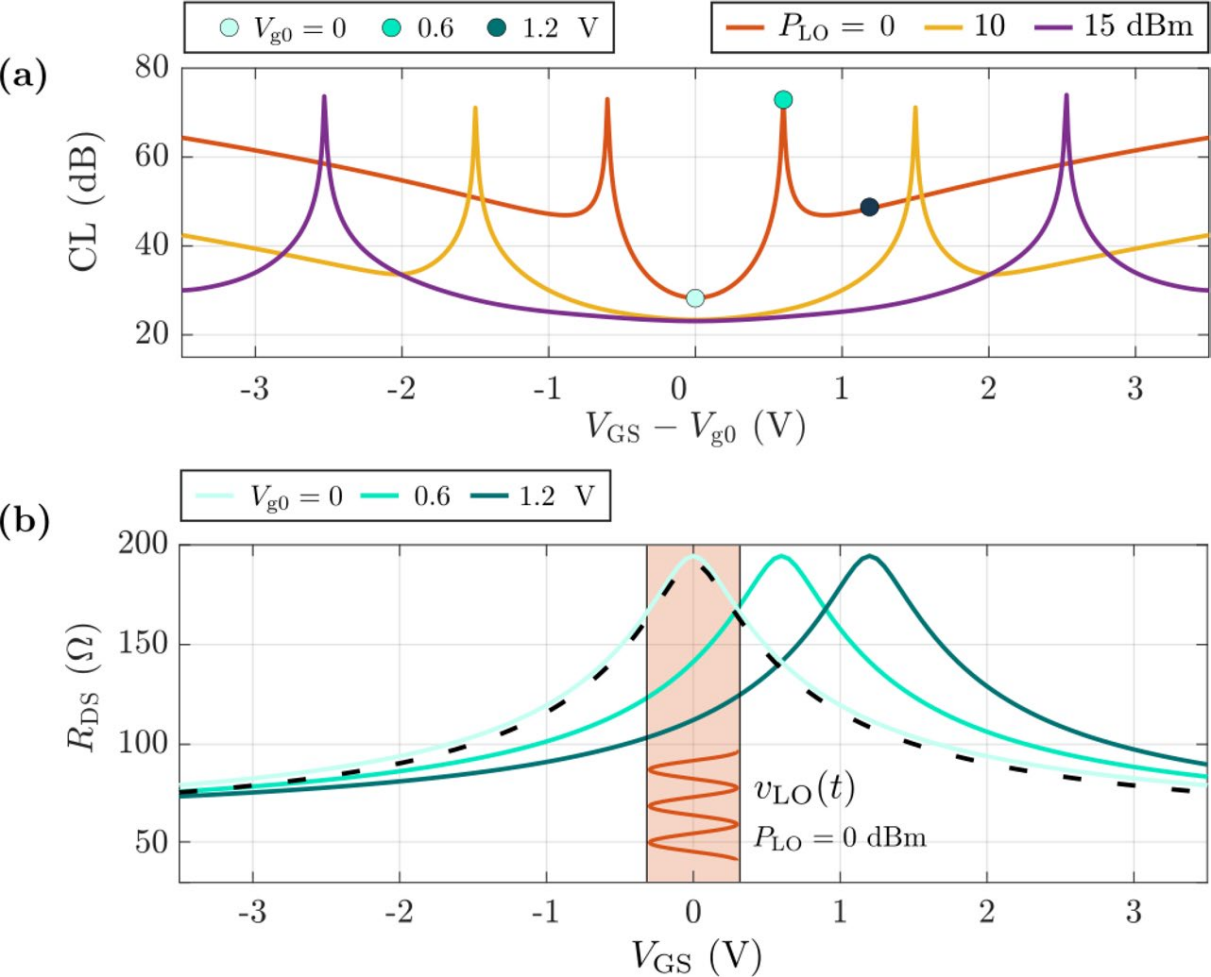
Study of the effect of ${V}_{\text{GS}}$ and ${V}_{\text{g0}}$ mismatching on mixing performance. **a** Conversion losses versus overdrive gate bias for different LO power levels. **b** ${R}_{\text{DS}}-{V}_{\text{GS}}$ characteristics for different ${V}_{\text{g0}}$ values; the time-varying LO input signal is depicted in orange, corresponding to ${P}_{\text{LO}}=0$ dBm

We first consider the effect of a shift in the ${V}_{\text{GS}}$ bias around ${V}_{\text{Dirac}}$, which in single-gated GFET operating at ${V}_{\text{DS}}=0\hspace{0.25em}\text{V}$ (as in the resistive mixer) corresponds to ${V}_{\text{Dirac}}={V}_{\text{g}0}$ [28]. Figure 8a shows CL as a function of ${V}_{\text{GS}}-{V}_{\text{g}0}$ for different local oscillator power levels. Minimum CL are obtained when the gate is precisely biased at ${V}_{\text{g}0}$, as the LO signal, ${v}_{\text{LO}}\left(t\right)$, takes full advantage from the graphene quadratic response. This can be better appreciated in Fig. 8b where ${R}_{\text{DS}}-{V}_{\text{GS}}$ is represented for different values of ${V}_{\text{g}0}$ (corresponding to the blue circles in Fig. 8a) together with a sketch of a ${v}_{\text{LO}}\left(t\right)$ signal with an amplitude corresponding to ${P}_{\text{LO}}=0\hspace{0.25em}\text{dBm}$. As can be noted, CL strongly degrades as the ${V}_{\text{GS}}=0\hspace{0.25em}V$ point is shifted from the vertex of the *R-V* inverted parabola in Fig. 8b, i.e., when there is a large mismatch between ${V}_{\text{GS}}$ and ${V}_{\text{g}0}$. This leads to the deterioration of the symmetrical quadratic behavior of the transfer characteristic in the range of the LO oscillator signal (red-shaded region in Fig. 8b), in such a way that the frequency doubling is not optimally achieved. In more detail, higher order harmonics different from the second order are generated, provoking a severe reduction of the down-converted IF signal output power. This results in an increment of the CL, as observed in Fig. 8a. Notably, the plateau in Fig. 8 where the CL remains small (e.g. below 30 dB) is larger for higher ${P}_{\text{LO}}$.

In other words, high ${P}_{\text{LO}}$ values make the GFET mixer more tolerant to gate bias mismatching in terms of CL. This is explained by the fact that a larger ${v}_{\text{LO}}$ amplitude eases the operation in the quadratic region of the GFET $I-V$ curve (i.e., within the graphene ambipolarity regime) for higher $\left|{V}_{\text{GS}}-{V}_{\text{g}0}\right|$ values. This is identified with a widening in the CL versus ${V}_{\text{GS}}-{V}_{\text{g}0}$ curves as ${P}_{\text{LO}}$ increases (cf. Figure 8a). This result gives critical insights on the tolerance needed for controlling the charge neutrality point (CNP) in GFETs for their ulterior application for subharmonic mixing depending on the available LO power.

The monotonic increase in CL with $\left|{V}_{\text{GS}}-{V}_{\text{g}0}\right|$ is interrupted (a discontinuity in the derivative of the CL is indeed observed) for $\left|{V}_{\text{GS}}-{V}_{\text{g}0}\right|$ values close to the amplitude of ${v}_{\text{LO}}$, i.e., when ${V}_{\text{Dirac}}$ leaves the range of ${v}_{\text{LO}}$ amplitudes. This behavior is related to the transition of the GFET channel from ambipolar to fully unipolar conduction. This is a remarkable result because it brings to light that graphene ambipolarity presents a very interesting nonlinear feature that can be exploited for nonlinear high-frequency applications [16], such as mixers and frequency multipliers [8]. The latter have already been demonstrated in the form of a frequency tripler [9] and quadrupler [10] by producing a mismatch between ${V}_{\text{GS}}$ and ${V}_{\text{Dirac}}$, showing that other higher order harmonics different from the first and second orders can be produced within the ambipolar regime. It is worth to note that, beyond the biases at which the CL get their maximum value, the unipolar conduction retains a nonlinear behavior, making subharmonic operation still possible. Certainly, that region results on much higher CL than the minimum value reached at ${V}_{\text{GS}}={V}_{\text{g}0}$, where the ambipolarity is fully exploited for frequency doubling.

**Fig. 9:**
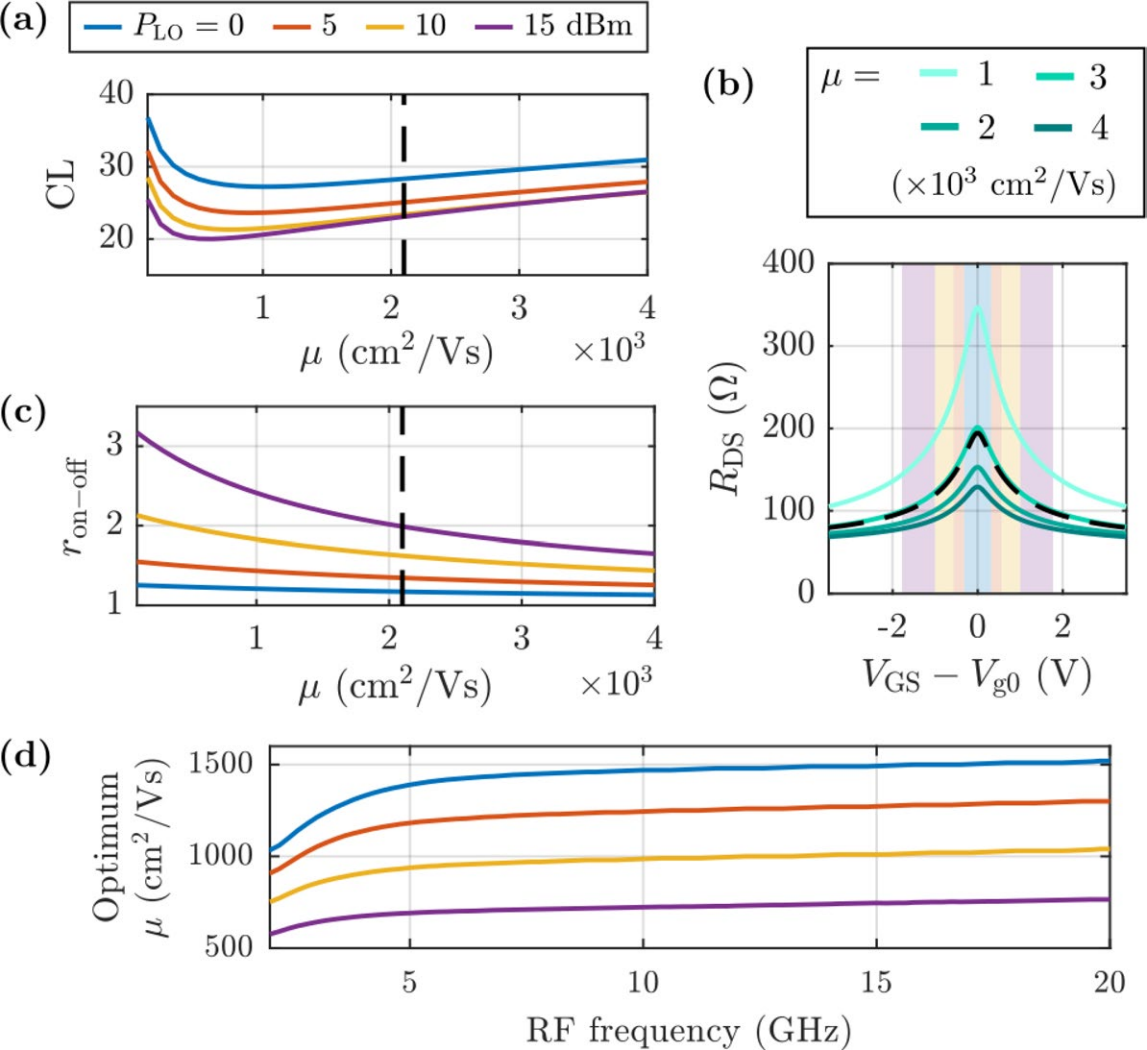
Study of the effect of $\mu$ on mixing performance. **a** Conversion losses for different LO power levels. **b** ${R}_{\text{DS}}-{V}_{\text{GS}}$ characteristics for different $\mu$ values. **c** On–off ratio calculated from the ${R}_{\text{DS}}-{V}_{\text{GS}}$ curves within the $\pm 3$ V gate voltage window. **d** Optimum mobility value reaching the minimum conversion losses as a function of the frequency of the RF signal. A black dotted line indicates the mobility value for the device under consideration (Table 1)

Next, the influence of the carrier mobility on the performance of the mixer is evaluated. Figure 9a shows the dependence of CL on the mobility for different LO power levels. In contrast to the conventional trend observed in transconductance mixers, in which ${g}_{\text{m}}$ and consequently CL are limited by $\mu$ [37], losses in a GFET subharmonic mixer have a non-monotonic behavior with $\mu$, giving rise to an optimum value dependent on the power of the local oscillator.

In order to explain this behavior, it is worth to pay attention to a well-known performance metric in conventional resistive mixers, i.e. the on–off resistance ratio ${r}_{\text{on}-\text{off}}={R}_{\text{DS},\text{off}}/{R}_{\text{DS},\text{on}}$ [38], which has been extended to contemporary realizations of GFET subharmonic designs [18, 19, 39–41]. Figure 9b shows the GFET DC *R-V* characteristics for varying $\mu$, exhibiting monotonic increases of both ${R}_{\text{DS},\text{off}}$ and ${R}_{\text{DS},\text{on}}$ with decreasing mobility. The on–off resistance ratio is then computed from the *R-V* curves, resulting in the trends observed in Fig. 9c. It must be noted that ${R}_{\text{DS},\text{on}}$ and ${R}_{\text{DS},\text{off}}$ vary with ${P}_{\text{LO}}$ as the LO voltage oscillation ranges a different *R-V* window (cf. Figure 8a). As can be observed, higher $\mu$ values result in a considerable reduction of ${r}_{\text{on}-\text{off}}$, explaining the strong deterioration in CL seen in Fig. 9a for the high carrier mobility region $\left(\mu \gtrsim 1000\hspace{0.25em}{\text{cm}}^{2}/\text{Vs}\right)$. Nevertheless, the higher ${r}_{\text{on}-\text{off}}$ seen in Fig. 9c for low mobility values is not translated into a reduction in CL, which, in fact, experience a trend change, increasing for $\mu \lesssim 200$ cm ${}^{2}$/Vs and thus resulting in an optimum $\mu$ for minimum CL. This is due to the fact that the analysis of the CL based on the behavior of the DC ${r}_{\text{on}-\text{off}}$ relies on the assumption that the GFET mixer operation frequency is lower than the transistor cutoff frequency in the nonlinear regime, ${f}_{\text{T}}^{\text{NL}}\left(\propto \mu \right)$, which sets the operating frequency limit. A significant decrease in ${f}_{\text{T}}^{\text{NL}}$ is expected at very low $\mu$, meaning that a non-negligible portion of the LO power will be leaked through the capacitive couplings between the GFET terminals. This unavoidably leads to an increase in the CL, as the effective power provided by the LO contributing to the mixing is reduced. In this regard, Fig. 9d depicts the optimum GFET mobility for minimizing CL as a function of the RF frequency, where higher operating frequencies require a rise of the optimal mobility. In addition, it must be highlighted that the larger the LO power, the lower the value of the optimum $\mu$, indicating that the cutoff frequency for this nonlinear application increases with ${P}_{\text{LO}}$. This is beneficial as a lower value of the optimum $\mu$, allows for higher resistance ratios that improve losses. Thus, it can be concluded that for increasing ${P}_{\text{LO}}$, the mobility region where ${f}_{\text{T}}^{\text{NL}}$ limits the mixer behavior, i.e., for mobilities lower than the optimum $\mu$, diminishes; and consequently, the region dominated by ${r}_{\text{on}-\text{off}}$ increases, i.e., for mobilities higher than the optimum $\mu$.

**Fig. 10:**
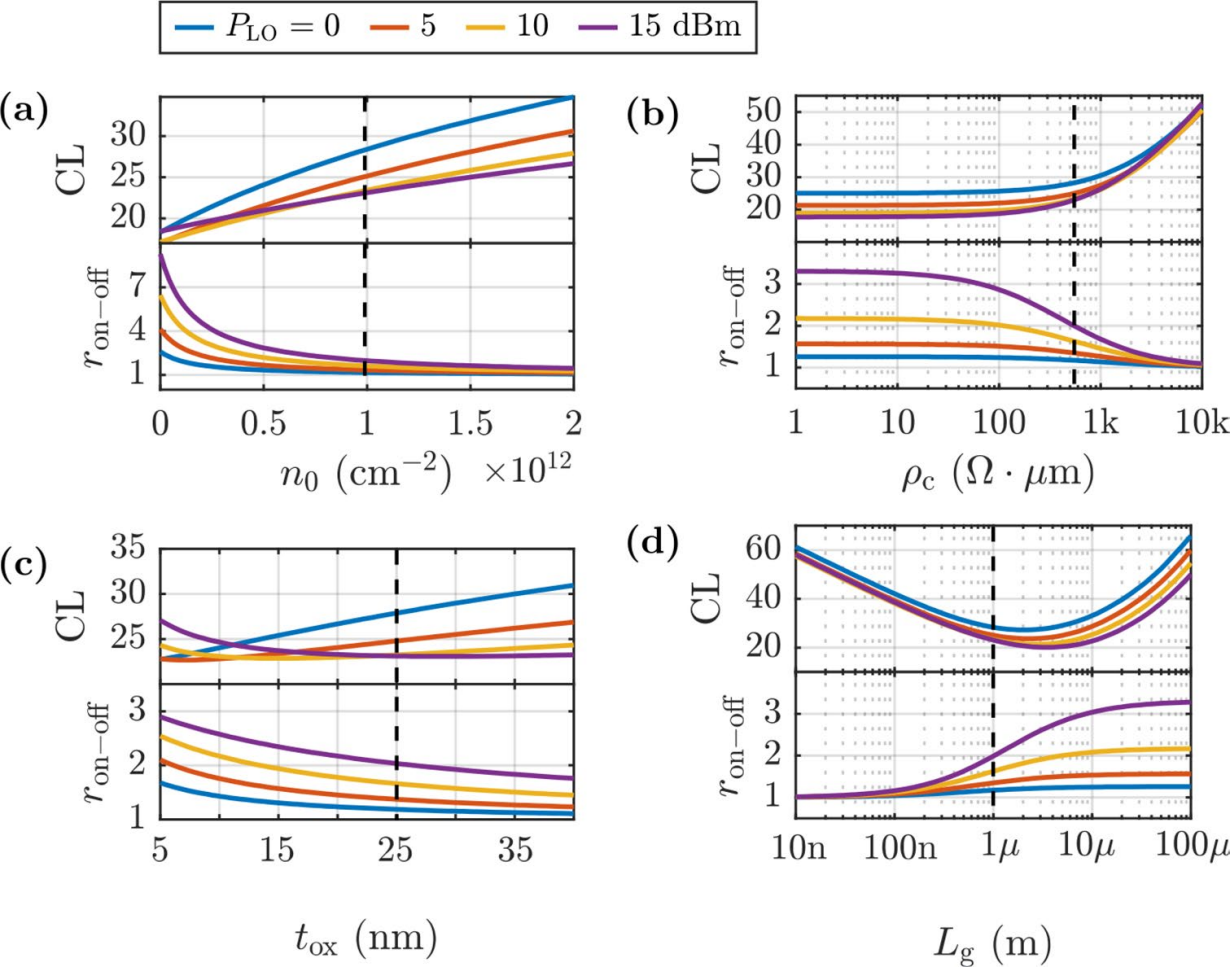
Evaluation of CL (top) and ${r}_{\text{on-off}}$ (bottom) as a function of **a** residual carrier density (${n}_{0}$); **b** contact resistivity (${\rho }_{\text{c}}$); **c** oxide thickness (${t}_{\text{ox}}$); **d** gate length (${L}_{\text{g}}$). Black dotted lines highlight the value corresponding to the device under consideration (Table 1)

The impact of the technological parameters is concluded with the study of the residual carrier density and the contact resistivity on both CL and ${r}_{\text{on}-\text{off}}$, evaluated in Fig. 10a-b. As can be seen, the increase of ${n}_{0}$ and ${\rho }_{\text{c}}$, portrays a continuous upward trend in losses, which aligns with the concurrent downward trend observed for ${r}_{\text{on}-\text{off}}$, meaning that this metric is able to anticipate the effect of the variability of these parameters.

Finally, we evaluate the role of the GFET geometrical parameters on the mixer performance. In particular, Fig. 10c–d shows the influence of the oxide thickness and gate length on CL and ${r}_{\text{on}-\text{off}}$. As can be noticed, while ${r}_{\text{on}-\text{off}}$ increases monotonically with decreasing ${t}_{\text{ox}}$ and increasing ${L}_{\text{g}}$, as well as with ${P}_{\text{LO}}$, the behavior is more irregular for CL. Losses follow a non-monotonic trend with these parameters, presenting a minimum which is shifted by ${P}_{\text{LO}}$. This evidences that the CL behavior cannot be predicted based solely on the analysis of ${r}_{\text{on}-\text{off}}$. Indeed, as explained before, if the operating frequencies involved in the mixing process are much higher than the transistor cutoff frequency, with qualitative trends of ${f}_{\text{T}}^{\text{NL}}\propto {t}_{\text{ox}}$ and ${f}_{\text{T}}^{\text{NL}}\propto {L}_{\text{g}}^{-2}$ [37], the AC large signal behavior becomes essential. In this regard, an increase in oxide thickness leads, in principle, to higher CL due to a decrease in the on–off ratio (Fig. 10c). However, decreasing ${t}_{\text{ox}}$ rises the oxide capacitance ${C}_{\text{ox}}$, therefore causing part of the LO power to leak through the capacitive coupling rather than contributing to the modulation of ${R}_{\text{DS}}$. In fact, the same effect would occur if the operating frequency of the circuit were significantly increased, as this would also reduce the impedance associated with the oxide, allowing for greater filtering of the LO power toward the output.

The non-monotonic behavior of CL vs. gate length, illustrated in Fig. 10d, can also be qualitatively split in two regimes. For short gate lengths, up to the optimum gate length reaching the minimum CL, the rising resistance ratio induces a negative slope in the CL vs. ${L}_{\text{g}}$ curve, indicating potential enhancement of mixer performance through the design of longer gates. The reversal in this trend, observed for larger ${L}_{\text{g}}$ values, is attributable once again to the device nearing the limit given by its cutoff frequency when operating as mixer. In addition, higher optimum ${L}_{\text{g}}$ is observed due to the higher ${f}_{\text{T}}^{\text{NL}}$ with increasing LO power.

## Conclusion

A systematic design approach was developed for a single-GFET subharmonic mixer, ensuring the preservation of a straightforward topology featuring zero-drain bias, which renders the circuit a promising candidate for future ubiquitous frequency-conversion IoT systems. In more detail, we presented a novel circuit design approach, including the selective LO filtering using a quarter-wave resonator to optimize the rejection of LO-related spurious components, and the simultaneous conjugate matching strategy using a quasi-linear approximation achieving very low IF and RF reflection coefficients. These techniques can be directly applied to any other subharmonic mixer topology, contributing to the advancement of the state of the art for these circuits.

Furthermore, an in-depth examination of the GFET technology drivers and its correlation with the mixer performance has revealed the trade-off between a wider drain-to-source resistance range (i.e., the on–off ratio) and the overall increase in current resulting from higher carrier mobility or shorter gates, within the constraints imposed by the device dynamic response. Such findings mark a significant advancement in the present understanding of the GFET-based mixer behavior.

## Data Availability

The datasets generated during the current study are available from the corresponding author upon reasonable request.

## Appendix A

The expressions of optimum source and load reflection coefficients ${\Gamma }_{\text{MS}}$ and ${\Gamma }_{\text{ML}}$ that attain simultaneous conjugate matching in a two-port circuit are [36]:

7 $${\Gamma }_{\text{MS}}=\frac{{B}_{1}\pm \sqrt{{B}_{1}^{2}-4{\left|{C}_{1}\right|}^{2}}}{2{C}_{1}}$$

8 $${\Gamma }_{\text{ML}}=\frac{{B}_{2}\pm \sqrt{{B}_{2}^{2}-4{\left|{C}_{2}\right|}^{2}}}{2{C}_{2}}$$

where.

9 $${B}_{1}=1+{\left|{S}_{11}\right|}^{2}-{\left|{S}_{22}\right|}^{2}-{\left|\Delta \right|}^{2}$$

10 $${B}_{2}=1+{\left|{S}_{22}\right|}^{2}-{\left|{S}_{11}\right|}^{2}-{\left|\Delta \right|}^{2}$$

11 $${C}_{1}={S}_{11}-\Delta {S}_{22}^{*}$$

12 $${C}_{2}={S}_{22}-\Delta {S}_{11}^{*}$$

13 $$\Delta ={S}_{11}{S}_{22}-{S}_{21}{S}_{12}$$
